# Relationship between structural abnormalities in the cerebellum and dementia, posttraumatic stress disorder and bipolar disorder

**DOI:** 10.1590/S1980-57642012DN06040003

**Published:** 2012

**Authors:** Leonardo Baldaçara, João Guilherme Fiorani Borgio, Célia Araújo, Fabiana Nery-Fernandes, Acioly Luiz Taveres Lacerda, Walter André dos Santos Moraes, Maria Beatriz Marcondes Macedo Montaño, Marlos Rocha, Lucas C. Quarantini, Aline Schoedl, Mariana Pupo, Marcelo F. Mello, Sergio B. Andreoli, Angela Miranda-Scippa, Luiz Roberto Ramos, Jair J. Mari, Rodrigo Affonseca Bressan, Andrea Parolin Jackowski

**Affiliations:** 1 Federal University of São Paulo, SP, Brazil.; 2 Federal University of Tocantins, TO, Brazil.; 3 Federal University of Bahia, BA, Brazil.

**Keywords:** cerebellum, neuroimaging, mental disorders, risk factors, comparative study

## Abstract

New evidence suggests that the cerebellum has structural and functional abnormalities in psychiatric disorders.

**Objective:**

In this research, the goal was to measure the volume of the cerebellum and its subregions in individuals with psychiatric disorders and to relate these findings to their symptoms.

**Methods:**

Patients with different degrees of cognitive impairment (Epidemiology of the Elderly - UNIFESP) and patients with post-traumatic stress disorder (PTSD) from population studies were analyzed. Also, patients with bipolar disorder from an outpatient clinic (Center for the Study of Mood and Anxiety Disorders, Universidade Federal da Bahia) were recruited for this study. All subjects underwent a 1.5T structural magnetic resonance scan. Volumetric measures and symptom measurements, by psychometric scales, were performed and compared between patients and controls.

**Results:**

The cerebellum volume was reduced in patients with cognitive impairment without dementia and with dementia, in patients with PTSD, and in patients with bipolar disorder compared to controls. In dementia and PTSD, the left cerebellar hemisphere and vermis volume were reduced. In bipolar disorder, volumes of both hemispheres and the vermis were reduced. In the first two studies, these cerebellar volumetric reductions correlated with symptoms of the disease.

**Conclusion:**

The exact nature of cerebellar involvement in mental processes is still not fully understood. However, abnormalities in cerebellar structure and its functions have been reported in some of these diseases. Future studies with larger samples are needed to clarify these findings and investigate whether they are important for treatment and prognosis.

## Abstract (pt)

Novas evidências sugerem que o cerebelo apresenta alterações estruturais e funcionais nos transtornos psiquiátricos.

**Objetivo:**

Medir o volume do cerebelo e de suas sub-regiões em indivíduos portadores de transtornos psiquiátricos e relacionar tais achados aos sintomas.

**Métodos:**

Foi realizada a identificação de pacientes com diferentes graus de prejuízo cognitivo proveniente de um estudo populacional (Epidemiologia do Idoso - UNIFESP), pacientes com transtorno do estresse pós-traumático proveniente de outro estudo populacional e portadores de transtorno bipolar proveniente de um ambulatório especializado (Universidade Federal da Bahia). Todos os sujeitos foram submetidos à ressonância magnética estrutural de 1.5T. As medidas de volume, assim como os sintomas medidos por escalas psicométricas foram comparadas entre pacientes e controles.

**Resultados:**

Foi observado que o volume do cerebelo está reduzido nos portadores de prejuízo cognitivo sem demência e com demência, no transtorno do estresse pós-traumático e no transtorno bipolar quando comparados aos controles. Na demência e no transtorno do estresse pós-traumático o volume do hemisfério cerebelar esquerdo e do vérmis estão reduzidos. No transtorno bipolar os volumes de ambos os hemisférios e do vérmis estão reduzidos. Nos dois primeiros estudos estas reduções correlacionaram com os sintomas.

**Conclusão:**

A natureza exata do envolvimento do cerebelo nos processos mentais ainda não é compreendida. Entretanto, anormalidades na estrutura cerebelar e em suas funções têm sido relatadas em algumas dessas doenças. Pesquisas futuras, com amostras maiores, ainda são necessárias para esclarecer tais achados e investigar se são importantes para o tratamento e prognóstico.

## INTRODUCTION

The term cerebellum comes from Latin, which means "little brain". This is similar in structure to the brain and located in the posterior fossa of the skull, underlying the occipital lobe, posterior to the brainstem.^1,2^ This similarity to the brain is mainly because it is formed by two hemispheres (right and left) and cortical (gray matter) matter around a center of white matter, which in turn has gray matter nuclei. Within the hemisphere there is a central worm-shaped part called the vermis.^1^

For many years, functions related only to movement, gait, posture, and balance were attributed to the cerebellum.^2^ However, some studies have suggested possible involvement of the cerebellum in cognition, emotion processing and behavior.^3-5^ According to this perspective, the cerebellum is believed to exert a regulatory function that enhances and supplements other brain functions, through direct and indirect circuits.^2^

Moreover, different sources of evidence have suggested that the cerebellum may be altered in many psychiatric disorders, including schizophrenia,^6^ bipolar disorder (BD),^7^ unipolar depression,^8^ anxiety,^9^ and attention deficit hyperactivity disorder.^10^ In some reports, cerebellar deficits were described as isolated findings, without any relationship with clinical history whereas others show evidence that the cerebellum is a relevant brain structure possibly related to a range of psychopathological manifestations.^2^

Previous studies have reported cerebellar abnormalities in dementia.^11-13^ In neuropathological studies, neuronal shrinkage and loss are well known changes that accompany Alzheimer's Disease (AD) and are most notable in the temporoparietal neocortex, limbic system, and some neuronal groups such as the *locus coeruleus* of the brain stem and the basal nucleus of Meynert in the basal forebrain.^12^ Morphological studies, conversely, have focused on the traditional neuropathological hallmarks of AD, such as amyloid plaques, neurofibrillary tangles, and amyloid angiopathy,^12^ while little has been reported on neuronal loss and other structural changes in AD.^11^ Only one structural MRI study has previously demonstrated that the posterior cerebellar lobes are smaller in AD patients when compared to healthy controls, and that this atrophy is associated with poorer cognitive performance in AD subjects, but not in MCI patients.^13^

Furthermore, the view that the cerebellum is involved in the experience and regulation of emotions was posited more than half a century earlier^14,15^ and cerebellar findings have also been reported in BD. Cerebellar atrophy in mania^16-19^, cerebellar gray matter reduction^20^, and smaller vermal subregions V2 and V3 (posterior-inferior region) are the main findings in BD patients^7,21,22^. More recently, it has been postulated that the cerebellum plays a role in posttraumatic stress disorder (PTSD), and some studies have observed altered functioning of the left cerebellar hemisphere^23^ and vermis^24,25^ in PTSD patients. This evidence suggests a possible role for this structure in the pathophysiology of the disease. To a lesser extent, cerebellar, superior temporal gyrus, and pituitary differences have been reported in maltreated children and adolescents with PTSD.^9,26,27^

The aim of this article was to report the results of three studies that assessed cerebellar volume in a representative case of three different groups of mental disorders and explored a possible role of the cerebellum in their pathophysiological mechanisms. Finally, the implications of these findings and future directions are discussed.

## METHODS

**Participants.** To study cerebellar structure in cognitive impairment 96 subjects were selected from the outpatient clinic of Epidemiology of Elderly Study (EPIDOSO)^28^ coordinated by the Preventive Medicine Department of Federal University of São Paulo (UNIFESP). Subjects with AD (n=70) and cognitive impairment without dementia (n=16) were included, in addition to 10 subjects without cognitive complaints or functional impairment with a Mini-Mental State Examination (MMSE) score of 30. All subjects were classified with the Clinical Dementia Rating scale (CDR).^29^ Subjects were divided into five groups according to dementia severity (CDR score) as follows: 0=controls, 0.5=questionable or very mild impairment, 1=mild impairment, 2=moderate impairment, and 3=severe impairment. All subjects without cognitive complaints or functional impairment and an MMSE score of 30 were classified as CDR=0. The patients who met the diagnostic criteria proposed by Petersen et al. (1999) for MCI were included in the CDR=0.5 group.^30^ Alzheimer's diagnosis was reached according to the criteria of the National Institute of Neurological and Communicative Disorders and Stroke and the Alzheimer's Disease and Related Disorders Association (NINCDS-ADRDA).^31^ For more details see Baldaçara and Borgio et al. (2011).^32^

To assess cerebellar structure in posttraumatic stress disorder (PTSD) subjects were selected from the Violence Project (PROVE) of the Department of Psychiatry of UNIFESP. Forty-two PTSD cases (patients) and 42 resilient matched controls (individuals exposed to one or more traumatic events after the age of 18 who had not developed PTSD) were included, identified through an epidemiological survey that studied PTSD among the civilian population in the city of São Paulo.^33,34^ For more details see Baldaçara and Jackowski et al (2011).^35^

To study cerebellar structure in bipolar disorder (BD) subjects were selected from an outpatient clinic of the Federal University of Bahia (UFBA). A total of 48 patients with euthymic BD type I and 25 healthy controls were screened. Eight patients and three controls were excluded due to a history of neurological illness, head trauma, or inability to complete the MRI exam. The euthymia criterion was the attainment of scores on both the YMRS and HDRS of under 7 points. For more details see Baldaçara and Nery-Fernandes et al. (2011).^36^

**Measures.** Cerebellar volumes were correlated with clinical scales. For the cognitive impairment sample, the Structured Clinical Interview for DSM-IV (SCID),^37,38^ Clinical Dementia Rating scale (CDR),^29^ Mini-Mental State Examination (MMSE),^39^ and Functional Activities Questionnaire (FAQ) were applied.^40^ For the PTSD sample the SCID,^37,38^ Clinician-Administered PTSD Scale (CAPS),^41^ Beck Anxiety Inventory (BAI),^42^ Beck Depression Inventory (BDI),^43^ and Early Trauma Inventory (ETI) were used.^44^ For the BD sample the (SCID,^37,38^ Hamilton Depression Rating Scale (HDRS),^45^ Young Mania Rating Scale (YMRS),^46^ and Barratt Impulsiveness Scale (BIS-11) were employed.^47^

**Image acquisition and analysis**. Imaging data were acquired using GE 1.5T scanners. Structural MRI images were acquired using sagittal T1 acquisition series (TR=9.8 ms, TE=3.1 ms, flip angle=30º, NEX=1, matrix size=256×256, FOV=24 cm, thickness=1.0 mm), yielding 160 slices. Before scanning, a sagittal scout series (nine to eleven 5-mm-thick slices with a 1-mm interslice gap) was performed to determine image quality and clarity, as well as subject head position. Total brain volume was measured using voxel-based morphometry (VBM) methodology, which was implemented with the VBM Toolbox in SPM5 (dbm.neuro.uni-jena.de/vbm) as described previously.^48^ T1-weighted images were segmented in the original space. The sum of all voxel values within the segmented image approximates the total volume within the corresponding partition. Total brain volume was computed from the sum of gray and white matter volumes.^48^

The cerebellum was measured using region of interest (ROI) methodology. This method was chosen because it is more precise than other methods and residual heterogeneity can be correctly included in the analyses. First, the ROI tracing was performed using the Brains 2 semi-automated model, then manually corrected based on previous studies.^49-51^ The cerebellar hemispheres and vermal volumes were calculated by summing the areas of successive coronal slices after tracing the region of interest (ROI), and excluding cerebrospinal fluid (CSF). The measurements began as the cerebellum appeared laterally to the pons. The tentorium cerebella acted as the upper limit and the base of the cerebellum as the lower limit. The cisterna magna and transverse sinus were excluded.^49^ The last slice included was the one at which the cerebellum was no longer distinguishable from the transverse sinus or was no longer visible. The measurement of the vermis began at the slice where the anterior and/or inferior posterior lobes appeared. Measurements were made until the vermis was no longer visible.^49^ To evaluate inter-rater and test-retest reproducibility, two raters repeated the cerebellar measurements twice, at least one week apart, for 10 randomly selected subjects. Inter-rater reliability and test-retest was found to be high (ICC >0.96) for all cerebellar and vermal regions.

**Data analysis.** Data were codified and analyzed using the Statistical Package for Social Sciences (SPSS for Windows, version 15.0). Prior to conducting analyses, measures were examined for normality using the Shapiro-Wilk test. The level of significance was set at p < 0.05 using a two-tailed test. For detailed information about each statistical method see the specific articles.^32,35,36^

**Ethical issues.** All participants signed informed consent forms before participation. The study of cerebellar volume in subjects with cognitive impairment and in PTSD was approved by the Institutional Review Board (IRB) of the Federal University of São Paulo (CEP nº 0258/08 and CEP nº (1026/06). The study of bipolar subjects was approved by the Ethics Committee of the Federal University of Bahia (CEP nº16/2005).

## RESULTS

**Cerebellum in cognitive impairment by dementia.** Cerebellar hemisphere volumes were progressively lower in CDR 1, 2, and 3. The volume of the vermis, however, was found to be already reduced in the CDR 0.5 group, where this reduction was progressive with increasing CDR scores. For more details see Table 1.

**Table 1:** Intracranial volume, cerebral and cerebellar volumes in cm^3^ for each group.^32^

CDR	0 Mean (SD)	0.5 Mean (SD)	1 Mean (SD)	2 Mean (SD)	3 Mean (SD)	χ^2^/F	p	Duncan test (5%)
Intracranial volume	1722.19 (47.80)	1750.64 (99.12)	1728.96 (86.32)	1729.64 (78.39)	1747.19 (93.64)	0.334	0.854	0=0.5=1=2=3
Brain	596.09 (37.01); 1008.67 (95.30)	576.23 (42.24); 1034.39 (129.73)	536.44 (45.38); 1007.44 (114.49)	449.38 (87.39); 1019.38 (131.69)	418.91 (51.24); 1056.33 (66.36)	27.636	< 0.001	0 >0.5 >1 >2=3
Left temporal lobe	71.86 (5.45), 120.41(8.12)	70.23 (4.67); 120.65 (8.95)	58.46 (4.32); 98.18 (5.67)	51.45 (4.48); 88.62 (4.58)	47.96 (3.79); 83.92 (4.23)	124.041	< 0.001	0 >0.5 >1 >2 >3
Right temporal lobe	70.89 (5.22); 117.77 (8.56)	70.77 (5.55); 117.44 (6.78)	55.03 (4.46); 94.96 (7.19)	51.06 (4.65); 87.99 (7.68)	49.22 (5.67); 83.45 (7.41)	19.389	< 0.001	0=0.5 >1 >2=3
Left cerebellum	30.40 (2.47); 53.21 (5.17)	28.86 (2.38); 49.70 (4.22)	26.90 (2.16); 46.43 (3.43)	24.04 (1.58); 41.54 (2.76)	20.69 (1.63); 36.04 (1.83)	53.976	< 0.001	0 >0.5 >1 >2 >3
Right cerebellum	30.15 (2.10); 51.95 (4.31)	31.40 (2.44); 55.00 (5.78)	24.90 (2.27); 44.41 (3.22)	23.46 (1.58); 40.54 (2.76)	20.12 (1.60); 35.04 (1.84)	80.858	< 0.001	0=0.5 >1 >2 >3
Vermis	3.24 (0.48); 5.45 (0.82)	2.05 (0.31); 3.89 (0.54)	1.46 (1.84); 2.51 (0.33)	1.37 (0.29); 2.37 (0.50)	0.99 (0.27); 1.74 (0.50)	103.069	< 0.001	0 >0.5 >1=2 >3
Cerebellar anterior lobe	9.08 (1.49); 15.25 (4.84)	9.06 (1.64); 15.75 (2.38)	8.11 (1.93); 13.84 (1.38)	8.15 (2.46); 13.99 (3.23)	6.56 (2.31); 10.57 (3.11)	2.097	0.088	0=0.5 >1=2 >3
Cerebellar posterior lobe	53.85 (3.82); 88.42 (5.23)	51.22 (2.36); 84.35 (3.44)	42.53 (2.18); 76.55 (4.21)	39.95 (2.95); 69.72 (3.33)	34.85 (1.88); 60.35 (2.12)	17.563	< 0.001	0 >0.5 >1 >2 >3*

First volume: corrected by Intracranial volume. Second volume: Absolute volume.

There were significant correlations between all cerebellar regions and total scores on the MMSE, sub-tests, and the FAQ. However, when controlling for brain and temporal lobe volumes, few correlations remained significant. MMSE total scores correlated positively with volumes of the left temporal lobe, right cerebellum, and posterior cerebellar lobe. The MMSE language sub-test correlated positively with the left temporal lobe, right cerebellar hemisphere, posterior cerebellar lobe, and vermis. Attention correlated positively with the posterior cerebellar lobe and vermis volumes. FAQ scores correlated negatively with the left temporal lobe, right cerebellum, and vermis volumes. For more details see Table 2.

**Table 2:** Significant correlations between all cerebellar region volumes and scores on MMSE and FAQ*.

Test/Region		Statistical data
MMSE	Left temporal lobe	β =0.492, t=1.876, p=0.064
	Right cerebellum	β =0.0543, t=1.263, p<0.080
	Posterior cerebellar lobe	β =0.0627, t=1.774, p=0.058
MMSE language sub-test	Left temporal lobe	β =0.452, t=1.642, p=0.052
	Right cerebellar hemisphere	β =0.442, t=9.542, p < 0.020
	Posterior cerebellar lobe	β =0.422, t=1.284, p=0.062
	Vermis	β =0.380, t=4.520, p<0.018
MMSE attention sub-test	Posterior cerebellar lobe	β =0.365, t=2.252, p=0.044
	Vermis	β =0.260, t=2.747, p=0.007
FAQ	Left temporal lobe	β = -0.666, t= -3.196, p=0.002
	Right cerebellar hemisphere	β = -0.111, t= -1.736, p=0.086
	Vermis	β = -0.809, t= -2.884, p=0.005

* FAQ: Functional Activities Questionnaire.

Of the 26 subjects without dementia at baseline, 10 subjects (1 with CDR=0 and 4 with CDR=0.5) developed dementia after two years. There were no differences in intracranial volume, cerebral volume, temporal lobes, and right cerebellum between the two groups at baseline. In a logistic regression analysis, reduced left temporal lobe (OR=2.002, C.I.: 1.102-3.123, p=0.058), posterior cerebellar lobe (OR=1.402, C.I.: 1.004-2.903, p=0.068), and vermal volume (OR=1.480, C.I.: 1.024-2.803, p=0.042) were related to future dementia onset.

**Cerebellum in posttraumatic stress disorder**. There were no age (t= -1.74, p=0.08) or gender (χ^2^=1.42, df=1, p=0.34) differences between the PTSD and resilient control groups. The PTSD group presented significantly higher scores for history of early traumatic life events (t=2.49, p < 0.01) and all clinical variables as follows: re-experiencing symptoms (t=6.22, p < 0.01), avoidance and numbing symptoms (t=7.12, p < 0.01), hyperarousal symptoms (t=6.445, p < 0.01), total CAPS score (t=7.86, p < 0.01), anxiety (t=4.65, p < 0.01), and depressive symptoms (t=4.46, p < 0.01).

Of the PTSD patients, 81% (34 subjects; χ^2^=42.60, p < 0.01) presented comorbid major depressive disorder (MDD), 9.5% (4 subjects) presented panic disorder (PD), and 2.4% (1 subject) presented alcohol abuse disorder (AAD). Of the resilient controls, 78.6% (33 subjects) did not fulfill criteria for a psychiatric disorder, but 9.5% (4 subjects) presented MDD, 7.1% (3 subjects) PD, and 2.4% (1 subject) fulfilled the criteria for ADD. The PTSD sample included a heterogeneous range of traumatic experiences as follows: assault (38.1%), sexual and physical abuse (28.6%), sudden death of a loved one (19%), kidnapping (7.1%) and others (7.2%). The average duration of symptoms was 43 months (from 1 month to 18 years). The controls were also primarily victims of assault (35.7%), sexual and physical abuse (26.2%), sudden death of a loved one (16.7%), kidnapping (7.1%) and others (14.3%). Brain volume was smaller in the PTSD group (F=4.50, p=0.01). PTSD patients also had reduced left cerebellar hemisphere (F=2.55, p=0.04) and vermal volumes (F=13.49, p < 0.01) compared to resilient controls. For more details see Table 3.

**Table 3:** Brain measurement in PTSD patients and resilient controls.35

Volumes*	PTSD (n=42)			Controls (n=42)		F	p
	**Mean**	95% C.I.**		**Mean**	**95% C.I.^b^**		
Brain^+^	1096.27	1069.05-1123.48		1161.03	1133.82-1188.24	4.50	0.01
Total cerebellum^++^	101.93	98.97-104.88		105.52	102.55-108.46	1.45	0.22
Left cerebellar hemisphere^++^	50.55	48.89-52.20		53.61	51.95-55.26	2.55	0.04
Right cerebellar hemisphere^++^	51.38	49.89-52.88		51.89	50.40-53.39	1.01	0.41
Vermis^++^	7.04	6.70-7.39		8.74	8.34-9.08	13.49	<0.01

* Volumes are in cm^3^;

** Confidence Interval;

+ Adjusted for age, gender and comorbidity;

++ Adjusted for brain volume, age, gender and comorbidity.

In the PTSD group (but not in the control group), a significant negative correlation was observed between vermal volume and CAPS total score, re-experiencing symptoms, avoidance and numbing symptoms, hyperarousal symptoms, early traumatic life events, anxiety, and depressive symptoms. A negative correlation between left cerebellum volume and CAPS total score, re-experiencing symptoms, avoidance and numbing symptoms, hyperarousal symptoms, and anxiety was also observed . For more details see Table 4.

**Table 4:** Significant correlations between all cerebellar region volumes and clinical scale scores (only in PTSD group).

Scale/Region		Statistical data
Vermal volume	CAPS total score	β = -0.36, t= -3.44, p<0.01
	Re-experiencing symptoms	β = -0.34, t= -3.11, p<0.01
	Avoidance and numbing symptoms	β = -0.39, t= -3.65, p<0.01
	Hyperarousal symptoms	β = -0.25, t= -2.30, p=0.02
	Early traumatic life events	β = -0.32, t= -2.86, p<0.01
	Anxiety	β = -0.29, t= -2.544, p=0.013
	Depressive symptoms	β = -0.23, t= -2.04, p=0.045
Left cerebellar volume	CAPS total score	β = -0.43, t= -2.77, p=0.01
	Re-experiencing symptoms	β = -0.36, t= -3.30, p=0.02
	Avoidance and numbing symptoms	β = -0.37, t= -2.42, p=0.02
	Hyperarousal symptoms	β = -0.42, t= -2.74, p=0.01
	Anxiety	β = -0.32, t= -2.03, p=0.04

CAPS total score correlated positively with ETI - early trauma (β= -0.32, t= -3.02, p < 0.01). Moreover, when both variables were controlled in linear regression analysis, vermal volume continued to correlate negatively with CAPS total score (β= -0.34, t= -3.11, p < 0.01) and ETI (β= -0.25, t= -2.14, p=0.04).

**Cerebellum in bipolar disorder.** There was no age, gender or schooling difference among patients with BD and healthy controls. The mean number of suicide attempts was 1.6±0.8. There was no difference between patients with BD who exhibited suicidal behavior and those who did not in terms of age (F=0.72, p=0.49), gender (χ^2^=3.05, p=0.22), and years of schooling (F=0.26, p=0.77). BIS scores were higher in BD subjects with a history of suicide attempts (67.35±14.80) as compared to both those without attempts (58.34±8.64) and control subjects (58.52±9.05) (p=0.05). There were no differences in intracranial and brain volumes between the BD group and the healthy control group. The left cerebellum (t=5.58, p=0.02), right cerebellum (t=5.43, p=0.02) and vermis (t=20.20, p < 0.01) were smaller in the BD group. For more details see Table 5.

**Table 5:** Intracranial, brain and cerebellar volumes (cm^3^) in control, bipolar without and with suicide attempts groups.^36^

Variable	Control n=22	Bipolar disorder n=40	Bipolar disorder without history of suicide attempt (n=20)	Bipolar disorder with history of suicide attempt (n=20)	t / F	p
Intracranial volume*	1429.70±157.32	1399.37±153.19	1380.91±138.82	1417.82±167.87	0.55**, 0.55^+^	0.46**, 0.58^+^
Brain volume*	834.24±56.85	820.71±36.78	816.83±35.17	824.59±38.82	1.29**, 0.79^+^	0.26**, 0.46^+^
Left cerebellum*	44.31±7.51	40.18±6.03	40.85±5.53	39.50±6.57	5.58**, 2.97^+^	0.02**, 0.06^+^
Right cerebellum*	45.40±7.48	41.15±6.51	41.80±5.29	40.50±7.62	5.43**, 2.86^+^	0.02**, 0.06^+^
Vermis	5.62±0.81	4.76±0.66	4.97±0.71	4.56±0.55	20.20**, 12.19^+^	<0.01**^,+^

* Mean±SD , cubic centimeters (cm^3^);

** Controls versus bipolar (t-test, p value);

+ Controls versus bipolar with and without suicide attempts (F test; p value).

There was no correlation between vermal and cerebellar hemisphere volumes and BIS, number of suicide attempts, or number of mood episodes (manic, depressive or mixed episodes).

## DISCUSSION

The evidence of structural alterations in cerebellum volume in neuropsychiatric disorders is not novel. However, few studies have assessed cerebellar morphology in well-characterized samples and related this to psychopathology. Moreover, there was no previous research that related early trauma to cerebellar volume reduction in any psychiatric disorders.

In past studies, the cerebellum has only been implicated in movement, gait, posture, and balance.^2^ However, recent studies suggest significant interconnections between the cerebellum and prefrontal cortex subdivisions related to executive functioning (working memory, attention, inhibition of behaviors, and decision making), verbal memory and language.^3,4,52,53^ Afferent projections from parietal, temporal, and occipital cortices, and the limbic system implicated in the integration of sensitive and sensory information, visuospatial organization, visual memory, and control of behavior and motivation have also been proposed.^2,54,55^ More recently, a timekeeping or "clock" function has been postulated for the cerebellar cortex and the inferior olive (the sole source of climbing fiber inputs to Purkinje cells) based on their unique microstructure and intrinsic rhythmic oscillatory properties.^55-57^ Xu (2006) proposed that the primary role of the inferior olive and the climbing fiber system in timing is to mediate the encoding of temporal information independent of motor behavior.^56^

The notion that the cerebellum is involved in the experience and regulation of emotions was posited more than half a century earlier,^14,58^ and intimate afferent and efferent connections to the brainstem and limbic system have provided neuroanatomical support.^14,58^ The cerebellum has monosynaptic projections not only to the hypothalamus, septum, hippocampus, amygdala, and basal ganglia, but also to the brainstem nuclei, where the cerebellar projections stimulate dopamine and noradrenaline release by innervating the substantia nigra and locus coeruleus.^14,58^

One of the first reports to relate the cerebellum to emotional experience involved a patient who reported unpleasant feelings after electrical stimulation of the dentate nucleus and superior peduncle.^58^ Furthermore, electrophysiological responses in several limbic structures, including the hippocampus, amygdala, and septum, were recorded following electrical stimulation of the fastigial portion of the deep cerebellar nuclei in mammals.^58^ Additional support for the connection between the cerebellum and emotions in humans is provided by reports of an emotionally disturbed patient who received electrical stimulation in the fastigial nucleus.^58^ It was found that electrical discharges induced by electric stimulation correlated with the patient's experience of anger and tension. Moreover, there is evidence that chronic stimulation of the vermis using implanted subdural electrodes can normalize behavior in severely emotionally (severe anxiety or depression) dysregulated patients.^14,58^

One result from these studies is highly important: cerebellar volume reduction related to early trauma. Although previous studies have demonstrated structural^49,59,60^ and functional^61,62^ cerebellar abnormalities in PTSD subjects, none have found a relationship between early-life traumatic experiences and cerebellar alterations in adulthood. The correlations between early-life trauma and CAPS with cerebellar volumes found in the current study suggest that both traumatic events and PTSD symptoms have an effect on cerebellar structure. However, it is not yet clear whether reduced brain regions (e.g., the cerebellum) represent antecedent vulnerability for developing PTSD upon exposure to a traumatic event or a consequence of PTSD symptoms. Spinelli et al. (2009)^63^ conducted a study to identify structural abnormalities that may predict increased risk of stress-related neuropsychiatric disorders. In this study, mother-reared Rhesus monkeys were compared to peer-reared offspring. An enlarged vermis, dorsomedial prefrontal cortex, and dorsal anterior cingulated cortex were found in peer-reared monkeys; however, there were no differences in the corpus callosum or the hippocampus.^63^ Comparing the present results with those from previous studies, we speculate that cerebellar hyperactivity is present during the first few months after the stress factor, and cerebellar volume reduction is a consequence of this chronic hyperactivity that appears later. Adverse childhood factors may lead to an increased risk for later PTSD.^64^ Evidence has suggested that the developing cerebellum is vulnerable to environmental insults, including those of a physical and psychological nature.^27,58,65^ Environmental insults encountered during childhood, such as exposure to toxic levels of lead,^66^ chronic irradiation,^67^ low birth weight,^68^ and neonatal exposure to dexamethasone,^69^ preferentially damage cerebellar structures. The dysregulation of the limbic-hypothalamic-pituitary-adrenal (LHPA) axis with elevated levels of corticotrophin releasing hormone (CRH) has been consistently reported in traumatized individuals.^27,70^ Adults with PTSD, maltreated children with symptoms of mood and anxiety disorders, children with PTSD secondary to maltreatment, and infant primate mothers all show this dysregulation.^27,70-72^

One hypothesis to explain PTSD is that childhood abuse acts as a severe stressor that unleashes a cascade of events that affect brain development.^27^ Adult animals submitted to a single prolonged episode of early maternal deprivation show stress-induced corticosterone responses.^73^ Maternal deprivation induces neuronal degeneration and astroglial abnormalities in the hippocampus and cerebellar cortex of neonatal rats. Maternal separation may impair learning and memory in adult males by altering normal developmental features in glucocorticoid receptor expression.^73^ Thus, children exposed to trauma (early trauma) may experience chronically elevated CRH during pituitary development. Elevated CRH may lead to pituitary hypertrophy, which may be most pronounced during puberty, due to trophic factors.^27^ Chronic exposure to CRH may result in the downregulation of pituitary CRH receptors over time. This downregulation may be an adaptive mechanism that regulates pituitary hypertrophy, as the resultant high cortisol levels would otherwise result in medical illness, and damage to the brain^27^ and cerebellum.^74^

There are several limitations of this study that should be considered. First, although the overall sample size of patients was reasonable for a study using neuroimaging data, the examined subgroups in multivariate analysis was relatively small. Thus, there was insufficient statistical power to examine all aspects of psychopathological phenomena in patients groups. Second, although our findings suggest that psychiatric disorders indicates more severe brain structural anomalies, our findings do not necessarily indicate that these findings are exclusive to dementia, PTSD and BD. Third, the cross-sectional design precluded the opportunity to examine cause and effect. Further, three studies had well characterized samples and two studies were conducted in a community sample; therefore, these results may not be generalizable to other care service settings.

The cerebellum contributes not only to control of voluntary movement, coordination, balance, gait and posture, but also cognitive functions (memory, attention, language and executive functions), besides the control of emotions and behavior. However, the exact nature of involvement of the cerebellum in higher mental processes is not yet fully understood. In this context, techniques for structural and functional neuroimaging have proven valuable tools to study the contribution of the cerebellum in cognition and its role in psychiatric disorders. Abnormalities in cerebellar structure and its functions have been reported in some psychiatric disorders. In subjects with cognitive impairment, it was observed that cerebellar volume is reduced and correlated with the loss of attention and language, even before the development of Alzheimer's dementia. These data relate to previous studies that showed cerebellar abnormalities and Alzheimer's, but also extend the findings to early-stage disease and characterize more precisely the cognitive impairments. The post-traumatic stress disorder was found to reduce the volume of the left cerebellar hemisphere, associated with symptoms of re-experiencing, avoidance, hyperactivity and anxiety. Although lesions in hemispheres and associated emotional changes have previously been described, there is no support in the literature to elucidate the reason for this lateralization. A reduction in the volume of the cerebellar vermis associated with the same symptoms, and depressive symptoms and trauma in early life was also observed. This finding corroborates the literature, which observed the relationship of posterior-inferior regions of the cerebellum (more precisely the vermis) with the control of emotions. Further, the data may extend to the relationship between early life events in the genesis of mental disorders, a theory much discussed in the literature, yet difficult to prove. In bipolar disorder patients, a reduced total volume of the cerebellum, the hemispheres and vermis was found compared with controls. A volume reduction in patients with the disease and a history of suicide was also observed, but, contrary to expectations based on literature data, no associations with symptoms of impulsivity, number of mood episodes or duration of disease were identified. In the latter disorder this raises the question: Does the reduced volume of the cerebellum reflect a relationship directly accompanying the course of the disease or constitute an early change that could predispose to disease? Future research is needed to investigate the importance of structural abnormalities in cerebellar patients with psychiatric disorders in more depth. This calls for research with larger samples, more detailed neuropsychological testing and neuroimaging combining structural and functional techniques (such as the volume of interest - VOI). Also, it is important to observe different groups in the same disorder, based on different forms of activation of the cerebellum during the same task. The 21^st^ Century holds much to be discovered. Perhaps a new molecule, a new cellular process, unique signal processing in neuronal networks or an as yet unknown principle of control may change current concepts and further understanding on how this structure regulates and complements the other nervous functions. The cerebellar system appears to be the key to such discoveries, especially to clarify the operation of alternative circuits and circuits of refinement.
